# High-Throughput Screening Assay for Convalescent Sera in COVID-19: Efficacy, Donor Selection, and Variant Neutralization

**DOI:** 10.3390/microorganisms12081503

**Published:** 2024-07-23

**Authors:** Krishna P. Kota, Ilya Trakht, Gavreel Kalantarov, David Ordonez, Jiayi Wei, Stephanie Trefry, Evia Bavari, Jenny Richardson, Rouzbeh Zamani, Christy Raney, Farooq Nasar, Bruce Daugherty, Seth Lederman, Sina Bavari

**Affiliations:** 1 Tonix Pharmaceuticals, Frederick, MD 21701, USA; krishna.kota@tonixpharma.com (K.P.K.); david.ordonez@tonixpharma.com (D.O.); jiayi.wei@tonixpharma.com (J.W.); eviabavari@gmail.com (E.B.); rouzbeh.zamani@tonixpharma.com (R.Z.); christy.raney@tonixpharma.com (C.R.); farooq.nasar@tonixpharma.com (F.N.); bruce.daugherty@tonixpharma.com (B.D.); seth.lederman@tonixpharma.com (S.L.); 2 Department of Medicine, Columbia University Medical Center, New York, NY 10032, USA; it8@cumc.columbia.edu (I.T.); gfk1@cumc.columbia.edu (G.K.)

**Keywords:** SARS-CoV-2, high-content imaging, convalescent sera

## Abstract

Convalescent sera, rich in pathogen-specific antibodies, offers passive immunity to patients with infectious diseases. Screening assays using convalescent sera are crucial for evaluating therapeutic efficacy, selecting suitable serum donors, and standardizing assays. They measure antibody levels, neutralizing potential, and specificity against viruses like SARS-CoV-2, ensuring therapeutic serum contains potent antibodies. Standardized procedures enable reliable results and wider adoption of serum therapy for COVID-19. We have developed a high-content image-based assay for screening convalescent sera against SARS-CoV-2 variants. Using various cell lines, we identified optimal candidates, employed immunofluorescence to visualize infected cells, and assessed neutralizing antibody efficacy. Screening convalescent sera for therapeutic potential identified neutralizing activity against SARS-CoV-2 variants. Dose–response analysis showed variable neutralizing activity, with some sera exhibiting broad neutralization. Additionally, we explored the synergy between neutralizing sera and β-d-N4-hydroxycytidine (NHC), an initial metabolite of molnupiravir. These assays enhance serum therapy’s benefits for COVID-19 treatment and aid in understanding neutralizing activity against SARS-CoV-2 variants, addressing viral challenges.

## 1. Introduction

SARS-CoV-2 is the coronavirus responsible for the global COVID-19 pandemic. It is a positive-sense single-stranded RNA virus that belongs to the family Coronaviridae and the genus Betacoronavirus. The virus is characterized by its distinct spike proteins, which enable it to bind to and infect human cells. The virus was first identified in December 2019 in Wuhan, China, and has since spread rapidly around the world, causing significant public health, social, and economic impacts [1]. The primary mode of transmission is through respiratory droplets and close contact between individuals. SARS-CoV-2 can lead to a wide range of symptoms, from mild to severe, including fever, cough, and shortness of breath. More severe cases can result in pneumonia, acute respiratory distress syndrome (ARDS), multi-organ failure, and death [2].

Efforts to control the spread of the virus have included widespread testing, contact tracing, quarantine measures, social distancing, the development and distribution of vaccines, and antiviral therapeutics. Vaccines have proven effective in reducing the severity of illness and preventing death, but the emergence of new variants with increased transmissibility and potential immune evasion has presented ongoing challenges [3].

Serum therapy, specifically convalescent serum therapy, involves the transfusion of serum from recovered COVID-19 patients into patients currently fighting the infection.

Serum therapy offers immediate passive immunity, particularly beneficial for high-risk individuals with compromised immune systems or those unable to mount an adequate response to vaccines [4]. This is crucial as it safeguards them from infection and severe symptoms. Additionally, it serves as a treatment option for severe COVID-19 cases, in contrast to vaccines, which primarily offer preventive protection. This is vital in saving the lives of those already infected. Moreover, for individuals unresponsive to vaccines due to age, pre-existing conditions, or other factors, serum therapy may provide an alternative means of acquiring immunity or managing the infection, ensuring widespread access to protection against the virus [5].

This study underscores the potential of serum therapy in addressing the complex challenges posed by the SARS-CoV-2 virus and its variants. We developed and optimized in vitro cell-based high throughput imaging assays for screening convalescent sera to identify suitable convalescent serum donors for use in serum therapy. Screening assays such as ours will (a) ensure that the convalescent sera contain sufficient levels of potent neutralizing antibodies to potentially provide therapeutic benefits [1]; (b) identify individuals with high levels of neutralizing antibodies [6] whose peripheral memory B cells may be a source for developing human monoclonal antibodies; and (c) establish standardized procedures and criteria for quickly screening and evaluating convalescent sera [7].

In this report, we describe a comprehensive approach to develop and optimize a cell-based high-content image assay for assessing the neutralizing activity of convalescent sera against SARS-CoV-2 infection. The assay was then used to assess the efficacy of potential antiviral treatments for COVID-19, screen a repertoire of COVID-19 convalescent serum samples, and assess the synergy of combination treatments for reducing SARS-CoV-2 infection while minimizing damage to host cells.

## 2. Materials and Methods

### 2.1. Identifying a Suitable Cell Line for Use in an Initial Anti-SARS-CoV-2 Neutralizing Antibody Imaging Assay

Our initial efforts focused on identifying the most suitable cell line for initial screening assays of convalescent sera. All cell lines were purchased from ATCC and maintained in a 5% CO_2_ humidified incubator at 37 °C using Eagle’s Minimum Essential Medium (EMEM) containing 10% heat-inactivated fetal bovine serum (FBS) and 1% non-essential amino acids (NEAAs). We tested four cell lines: Vero E6 cells (ATCC, Manassas, VA, USA, Cat# CRL-1586), Vero-TMPRSS2 (ATCC NR-54970), HeLa Ace2 cells (BPS Bioscience, San Diego, CA, USA, Cat# 79958), and A549-hACE2 cells (InVivoGen, San Diego, CA, USA, Cat# ant-1002).

Cells were cultured in EMEM supplemented with 10% FBS for the assay. Once the cells reached 90% confluency, they were trypsinized and counted using a Beckman Coulter Vi-CELL Blu Cell Viability Analyzer (Beckman Coulter, Indianapolis, IN, USA). Mock-infected wells served as negative controls, while SARS-CoV-2-infected wells served as positive controls. Cells were seeded (40 µL/well) at varying cell densities in black, clear bottom 384-well plates (Greiner BioOne, Monroe, NC, USA, Cat #781920) (imaging plates) using Integra’s pipetting robot, Assist-plus (Cat # 4505). Cells were subsequently infected with SARS-CoV-2 variants ((Alpha, BQ.1.1, or XBB (BEI Resources, Manassas, VA, USA, Cat# NR-52281,NR-58976, and NR58925, respectively)) at various time points and various multiplicities of infections (MOI) ranging from 0.125 to 1.0 then incubated and fixed with 4% paraformaldehyde (PFA) for 15–20 min. Cells were washed with phosphate-buffered saline (PBS), then 50 µL/well of blocking buffer (3% bovine serum albumin (BSA) and 0.1% Triton X-100 in PBS) was added, and the plates were incubated at 37 °C in a humidified 5% CO_2_ incubator for one hour. To detect viral infection, SARS-CoV-2 Nucleocapsid Antibody (Rabbit Mab, Cat: 40143-R001, Sino Biologicals, Houston, TX, USA) was used as the primary antibody (Ab). It was diluted 1:1000 in blocking buffer, added to the plates (50 µL/well), and incubated for one hour at 37 °C in a humidified 5% CO_2_ incubator. Cells were washed with 0.05% Tween in PBS. Goat anti-rabbit IgG conjugated with Alexa 488 was used as the secondary Ab (Invitrogen, Cat # A32731). It was diluted 1:2000 in blocking buffer and added to the plates (50 µL/well). The plates were incubated for 1 h at 37 °C in a humidified 5% CO_2_ incubator. Cells were stained with a 1:10,000 dilution of HCS CellMask™ Deep Red Stain (Invitrogen, Cat # H32721) in PBS (50 µL/well) and a 1:2000 dilution of the nuclear dye Hoechst 33342 (Invitrogen Cat # H3570) in PBS (50 µL/well) to visualize cell structures and nuclei, respectively. The infected cells were imaged using the Opera Phenix high-content imager (Perkin Elmer, Waltham, MA, USA). The appropriate cell density and MOI for infections were selected based on factors such as the number of cells, cell viability, and spatial distribution of cells for image analysis. The robustness of the optimized assay in the 384-well plate format was evaluated using the Z’-factor, which provides a measure of separation between positive and negative controls [8].

### 2.2. Development of a Cell Viability Assay in Vero-TMPRSS2 Cells with SARS-CoV-2 (Alpha, BQ.1.1, or XBB) Infection

Understanding the toxicity of SARS-CoV-2 enabled the development and optimization of an in vitro neutralization assay to quantify anti-SARS-CoV-2 neutralizing antibody activity. We developed a cell viability assay with SARS-CoV-2 (Alpha, BQ.1.1, or XBB) infection. Vero-TMPRSS2 cells are derived from a monkey kidney cell line that has been genetically modified to express human transmembrane serine protease 2 (TMPRSS2), a protease enzyme that cleaves the spike protein of SARS-CoV-2, which in turn facilitates viral entry into cells [9]. Vero-TMPRSS2 cells were maintained in a 5% CO_2_ humidified incubator at 37 °C using Eagle’s Minimum Essential Medium (EMEM) containing 10% heat-inactivated fetal bovine serum (FBS) and 1% non-essential amino acids (NEAAs).

In these assays, Vero-TMPRSS2 cells were seeded into imaging plates (3000 cells per well, 40 µL per well) using a Fisher Multidrop Combi tip (Model 24073290). The plates were incubated for 24 h at 37 °C in a humidified 5% CO_2_ incubator and then infected with SARS-CoV-2 variants (Alpha, BQ.1.1, or XBB). We compared several durations of infection: 24 h, 36 h, and 48 h, along with varying multiplicities of infection (MOI) ranging from 0 to 0.5. After each incubation period (24, 36, and 48 h), the cells were fixed with 4% PFA for 15–20 min, washed with PBS, then blocked with blocking buffer for one hour at 37 °C in a humidified 5% CO_2_ incubator. To detect viral infection, SARS-CoV-2 Nucleocapsid Antibody (Rabbit Mab, Cat: 40143-R001, Sino Biologicals, Houston, TX, USA) was used as the primary Ab. It was diluted 1:1000 in blocking buffer, added to the plates (50 µL/well), and incubated for one hour at 37 °C in a humidified 5% CO_2_ incubator. Cells were washed with 0.05% Tween in PBS. Goat anti-rabbit IgG conjugated with Alexa 488 was used as the secondary Ab (Invitrogen Cat # A32731). It was diluted 1:2000 in blocking buffer and added to the plates (50 µL/well). The plates were incubated for 1 h at 37 °C in a humidified 5% CO_2_ incubator. Cells were stained using a 1:10,000 dilution of HCS CellMask™ Deep Red Stain (Invitrogen, Cat # H32721) in PBS and a 1:2000 dilution of the nuclear dye Hoechst 33342 (Invitrogen Cat # H3570) in PBS to visualize cell structures and nuclei, respectively. The infected cells were imaged using the Opera Phenix^®^ Plus High-Content Screening System (Perkin Elmer, Waltham, MA, USA).

### 2.3. Development of an In Vitro Neutralization Assay to Quantitatively Measure Neutralizing Antibodies against SARS-CoV-2 and Primary Screening of Convalescent Sera

Our next step in developing a high-throughput assay for screening potential donors for COVID-19 convalescent serum therapeutic applications was to develop and optimize an in vitro neutralization assay based on cell viability to quantitatively measure the efficacy of anti-SARS-CoV-2 antibodies in convalescent sera. The objective was to perform a primary screen of the convalescent sera received from Columbia University Medical Center, New York, against all three SARS-CoV-2 variants to identify sera that demonstrated inhibition to viral infection. We first studied the effect of anti-viral therapeutics in the cell viability assay to identify the most effective treatment while minimizing damage to the host cells (Figure S1). This also allowed us to compare the activity of neutralizing anti-SARS-CoV-2 activity in convalescent sera to the known activity of anti-viral inhibitors.

#### Source of Convalescent Sera

A total of 162 convalescent sera samples were collected from volunteers at Columbia University Medical Center, New York, (internal IRB number AAAT0368).

Vero E6 and Vero-TMPRSS2 cells were each seeded into 384-well imaging plates as previously described and incubated at 37 °C in a humidified 5% CO_2_ incubator overnight. The following day, in a separate 384-well plate, convalescent sera and the reference inhibitor NHC were each serially diluted eight times at a 1:3 dilution, starting at concentrations of 1:20 and 30 uM, respectively, using the Integra Viaflo 96/384 (Integra Model 410000) for serial dilutions. A 5 µL volume of the SARS-CoV-2 variants were combined with 5 µL of each serially diluted reference inhibitor or serially diluted sera and 40 µL of media. The mixture was incubated at 37 °C in a humidified 5% CO_2_ incubator for one hour and then tested in triplicate; 50 µL/well was added to the 384-well imaging plates containing Vero E6 or Vero-TMPRSS2 cells. Mock-infected Vero E6 /TMPRSS2 were included in this experiment as negative controls. Wells infected with the virus only (no anti-viral inhibitor or anti-viral Ab) were included in this experiment as positive controls. After 48 h of viral infection incubation at 37 °C in a humidified 5% CO_2_ incubator, the plates were formalin-fixed and stained, as previously described, for high-content imaging. The multiplicity of infection (MOI) and duration of infection for each virus were optimized to achieve an infection rate of around 60%.

Due to the significant number of hits obtained in the primary screening, further testing was conducted on the convalescent sera at two lower concentrations, 1:200 and 1:2000, to evaluate the efficacy of the sera against all three variants of SARS-CoV-2: Alpha, BQ1.1, and XBB. The objective of these additional tests was to assess the effectiveness of the sera at lower concentrations prior to initiating dose–response studies.

### 2.4. Dose–Response Curve and Efficacy Assessment of Individual Convalescent Serum Samples and Pooled Convalescent Sera

Convalescent sera with strong neutralizing Ab activity against all three variants of SARS-CoV-2 (≥80% viability compared to the positive controls) in the primary screen were selected for this assessment. Additionally, sera were pooled from a combination of samples with ≥80% viability in the primary screen and assessed for efficacy.

#### Anti-Viral Reference Inhibitor

β-d-N4-hydroxycytidine (NHC) is a mutagenic ribonucleoside that can act as a broad-based antiviral agent. Mutagenic ribonucleosides are metabolized to the active ribonucleoside triphosphate form and concentrate in the genomes of RNA viruses during viral replication, with antiviral activity correlated to the level of mutagenesis in virion RNA. NHC has demonstrated effectiveness against various SARS-CoV-2 strains, including the Omicron variant, and has a high genetic barrier to resistance [10].

Vero-TMPRSS2 cells were seeded at a density of 3000 cells per well (40 µL/well) in a 384-well imaging plate. In a separate 384-well plate, convalescent serum samples (diluted 1:20 in cell culture media) were combined with the respective SARS-CoV-2 variants and incubated at 37 °C in a humidified 5% CO_2_ incubator for two hours. The reference compound, NHC, was added to the Vero-TMPRSS2 cells two hours prior to adding the virus–sera mixture to the cells. The virus–sera mixture was added to the Vero-TMPRSS2 cells in triplicate. The infection was allowed to proceed for 48 h at 37 °C in a humidified 5% CO_2_ incubator. Both the reference compound and the convalescent sera were tested in a 7-point dose–response curve assay, utilizing a 3-fold serial dilution starting with a 1:20 dilution. After 48 h of infection, the cells were washed once with 1X PBS. Subsequently, the cells were fixed and subjected to immunofluorescence assay (IFA) to visualize the corresponding viral antigen expression and assess cell viability, following the same procedure as described in the primary screen. Percentage cell viability was determined as in the primary screen. The data obtained from the image analysis of the dose–response curve assay were plotted and analyzed using the non-linear regression formula: log (inhibitor) vs. response variable slope (4 parameters) in GraphPad Prism 6.

### 2.5. Synergy Assay

In this study, we explored the domain of synergy studies for drug interactions, a vital area in pharmacology and therapeutics. We employed a meticulous experimental approach, weaving together dose–response curves, potent combination indices, and rigorous statistical analyses. This dynamic interplay allows us to meticulously investigate the potential for synergistic or antagonistic interactions between drugs, illuminating the hidden complexities of drug synergy [11].

A synergy assay was performed to evaluate the interaction of the reference anti-viral inhibitor NHC and the pooled convalescent serum and the impact on potency of the combination based on cell viability. The combination of NHC and pooled convalescent serum was evaluated for synergistic effects. A synergy study was conducted using a 10 × 10 matrix with 10 dilutions and 3-fold dilutions against all three SARS-CoV-2 variants in Vero-TMPRSS2 cells.

### 2.6. Image Acquisition and Analysis

The Opera Phenix^®^ Plus High-Content Screening System was used to collect and analyze high-content quantitative imaging data as described previously [12,13]. Briefly, the system was equipped with two exposure settings and either a 10× air or 40× water objective. Cell images were obtained using a 10× air objective with a numerical aperture of 0.3, in a confocal mode at a bin of 2. The distance between the objective and the specimen was 5.2 mm, and the field of view of the images was 1.29 × 1.29, with an effective resolution of 2.6 µm in the XY plane. Nine fields were captured in each well, covering more than 90% of the surface area of the well.

To ensure that there was no crosstalk between channels, images of virally infected cells and the nuclear dye channel were simultaneously captured, while those of the whole cell dye channel were obtained separately. The analysis of the acquired images was performed within the Opera Phenix environment, utilizing custom-designed scripts in the Harmony environment. This approach allowed us to conduct a comprehensive high-content quantitative analysis of the imaging data, which provided valuable insights into the cellular processes under investigation.

The whole cell stain HCS CellMask™ Deep Red Stain was used to identify the cells (shown as red staining), and the nuclear stain Hoechst was used to visualize the nuclei (shown as blue staining). To classify infected cells, a predefined criterion was applied, considering cells that displayed a green signal within well-defined cell boundaries and surpassed a predetermined threshold level. The “Merge” image represents the combined view of all three images. Multiple parameters were extracted from the images, encompassing viral signal intensities in the nucleus and cytoplasm, nuclear size, nuclear intensities, as well as texture properties of the cytoplasm and nuclei, among others. For the primary screening assays, the focus was solely on the total cell count and infected cell count for calculating the percentage of infected cells.

### 2.7. Plaque Reduction Neutralization Test (PRNT50)

All serum samples were heat-inactivated at 56 °C for 30 min. Samples were serially diluted 2-fold with a solution containing DMEM, 2% FBS, and gentamicin (50 μg/mL); mixed with an equal volume of 2000 PFU/mL of SARS-CoV-2 variant; and incubated for 1 h at 37 °C. Vero E6 cell monolayers in 6-well plates were then inoculated with 100 μL of the serum–virus mixture in triplicate. SARS-CoV-2 anti-sera or monoclonal antibodies were used as positive controls, and medium-only was used as a negative control. Plates were incubated at 37 °C with 5% CO_2_ for 3–4 days, fixed with 10% formalin, and stained with crystal violet as described above. PRNT 50 values were calculated and expressed as the reciprocal of the serum dilution yielding an 50% reduction (PRNT80) in the number of plaques.

### 2.8. Statistical Analysis and Data Normalization

To assess the statistical reliability of SARS-CoV-2 infection data, we calculated the Z’-factor using the equation:

1 − [(3σp + 3σn) / |μp − μn|],

where μp, σp, μn, and σn represent the means (μ) and standard deviations (σ) of positive (p) and negative (n) controls [8,14].

Mock-infected and SARS-CoV-2-infected Vero-TMPRSS2 cells were used as negative and positive controls, respectively. Initial infection percentages were normalized using mock-infected and SARS-CoV-2-infected cells, considered 100%, and the resulting values were subtracted from 100 to determine the percentage of inhibition. For primary screening, a Z’-factor greater than 0.5 was employed to quantify the suitability of the assay. The selection criteria for a hit (i.e., an antibody that inhibits virus infection) in the primary screening were set at greater than 50% inhibition of SARS-CoV-2 infection.

## 3. Results

### 3.1. Identification of a Suitable Cell Line for Use in an Initial Anti-SARS-CoV-2 Neutralizing Antibody Imaging Assay; Vero E6, Vero-TMPRSS2, HeLa-Ace2, and A549-Ace2

**Figure 1:**
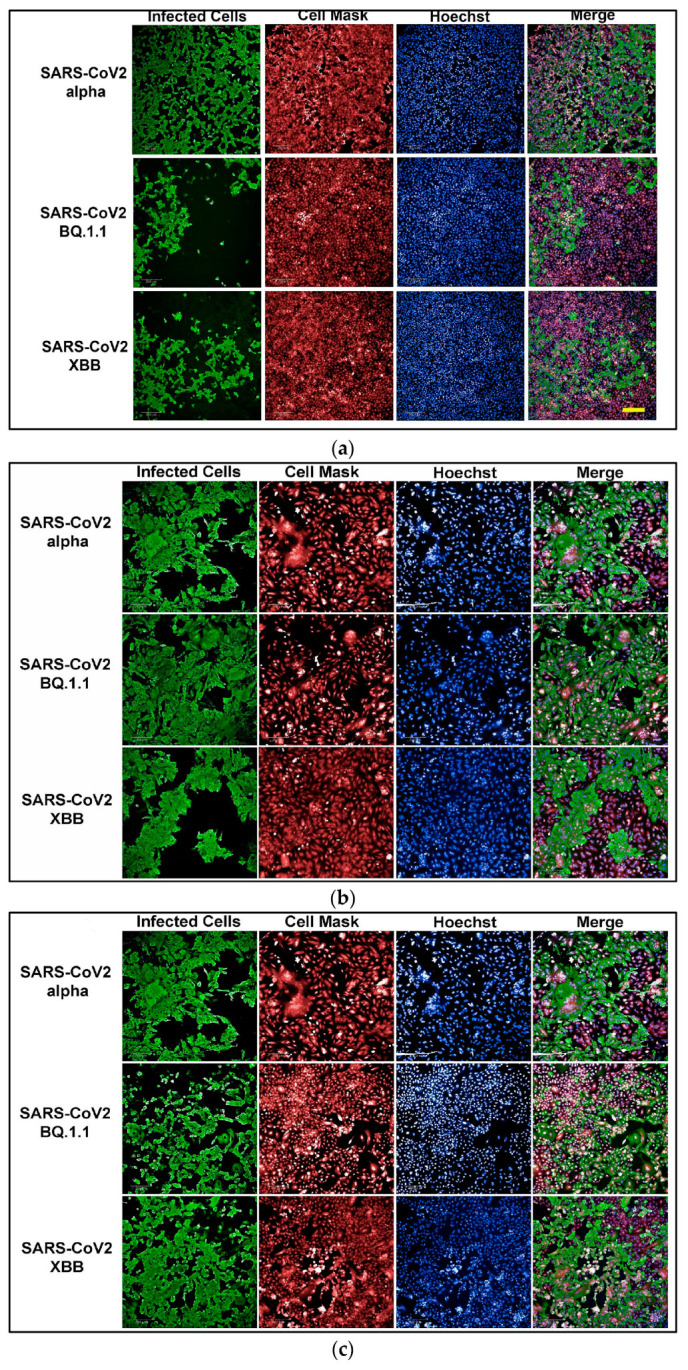

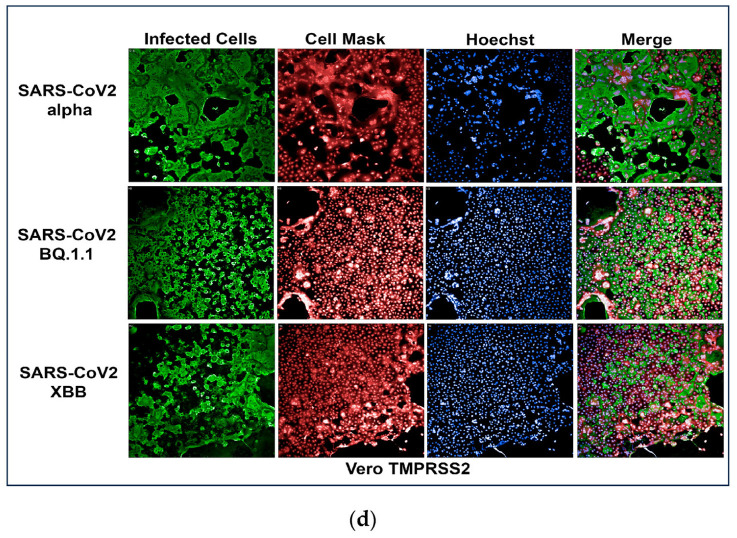
(**a**) High-content images of Vero E6 cells infected with the SARS-CoV-2 variants Alpha, BQ1.1, and XBB. In these high-content images, virus-infected cells are highlighted in green, while whole cells are stained in red using CellMask™, and nuclei are stained in blue using Hoechst dye. (**b**) High-content images of HeLa-ACE2 cells infected with the SARS-CoV-2 variants Alpha, BQ1.1, and XBB. In these high-content images, virus-infected cells are highlighted in green, while whole cells are stained in red using CellMask, and nuclei are stained in blue using Hoechst dye. (**c**) High-content images of A549-ACE2 cells infected with the SARS-CoV-2 variants Alpha, BQ1.1, and XBB. In these high-content images, virus-infected cells are highlighted in green, while whole cells are stained in red using CellMask™, and nuclei are stained in blue using Hoechst dye. (**d**) High-content images of Vero-TMPRSS2 cells infected with the SARS-CoV-2 variants Alpha, BQ1.1, and XBB. In these high-content images, virus-infected cells are highlighted in green, while whole cells are stained in red using CellMask™, and nuclei are stained in blue using Hoechst dye. Scale bar: 200 µm.

To develop an assay for SARS-CoV-2 infection, we incorporated high-content imaging (HCI). Four cell lines were tested, including Vero E6 (Figure 1a), Vero-TMPRSS2 (Figure 1d), HeLa-ACE2 (Figure 1b), and A542-ACE2 (Figure 1c). Vero-TMPRSS2 cells were found to be more permissive to SARS-CoV-2 infection and easier to culture, making them the most suitable for initial screening assays.

### 3.2. Development of a Cell Viability Assay in Vero-TMPRSS2 Cells with SARS-CoV-2 (Alpha, BQ.1.1, or XBB) Infection

A specific seeding density of 3000 cells per well was established for the SARS-CoV-2 infection HCI assay. A specific MOI was selected depending on the variant being studied, as shown in Table 1 below. Along with a 48 h incubation period, this carefully chosen combination resulted in infection rates of 60% or higher.

The Z’-factor was calculated using the average and standard deviations of the percent infection in the positive and negative controls, as described in the Methods Section. The experiment was performed in triplicate on three separate days, resulting in a calculated Z’-factor of 0.9. A Z’-factor greater than 0.5 indicates a statistically reliable separation between positive and negative controls.

**Figure 2:**
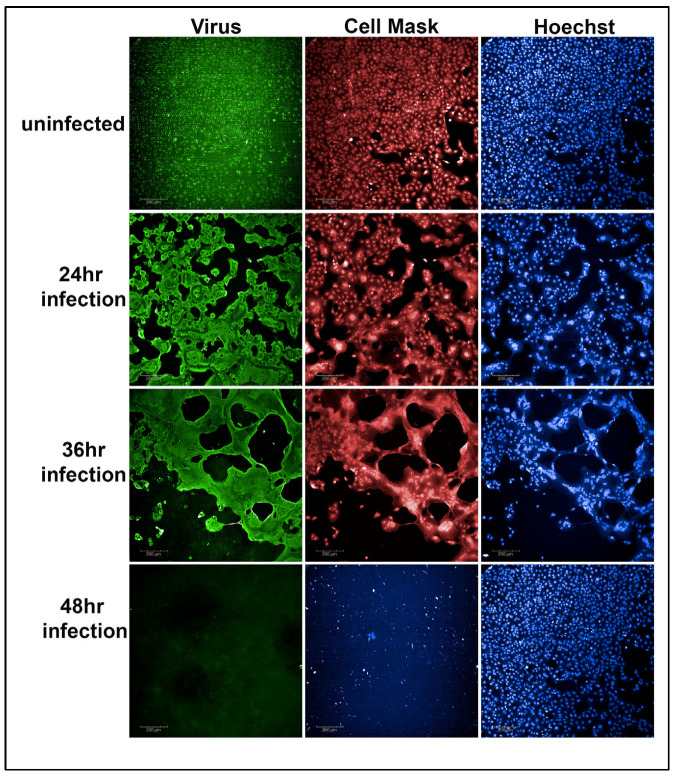
Representative example of Vero-TMPRSS2 cells, without infection, after 24, 36, and 48 h of virus infection with an MOI of 0.37. After 48 h of viral infection, the viability of Vero-TMPRSS2 cells was completely lost (48 h panel 2 figure) compared to the uninfected Vero-TMPRSS2 cells (48 h panel 3 figure).

The normalized percentage of cell viability served as a quantitative measure for evaluating the potency of the tested antibodies in neutralizing SARS-CoV-2 variants. Figure 2 demonstrates the results obtained after a 48 h SARS-CoV-2 Alpha variant infection with an MOI of 0.37 in Vero-TMPRSS2 cells. Notably, the 48 h time point and MOI combination resulted in a complete loss of cells. Therefore, 48 h infections with a 0.37 MOI were selected for use in the primary screen of convalescent sera.

### 3.3. Development of an In Vitro Neutralization Assay to Quantitatively Measure Neutralizing Antibodies against SARS-CoV-2 and Primary Screening of Convalescent Sera

We conducted a high-throughput primary screening assay to identify potential donors of COVID-19 convalescent sera. This involved optimizing an in vitro neutralization assay with a focus on cell viability to quantitatively measure the efficacy of anti-SARS-CoV-2 antibodies in convalescent sera.

In the study, we obtained 162 convalescent sera samples from Columbia University Medical Center. Vero-TMPRSS2 cells were seeded into 384-well imaging plates and incubated overnight. Each convalescent serum sample, along with NHC (an FDA-approved treatment for COVID-19), was screened in triplicate and combined with SARS-CoV-2 variants. The mixture was then added to cells, followed by a 48 h incubation period to allow for viral infection.

**Figure 3:**
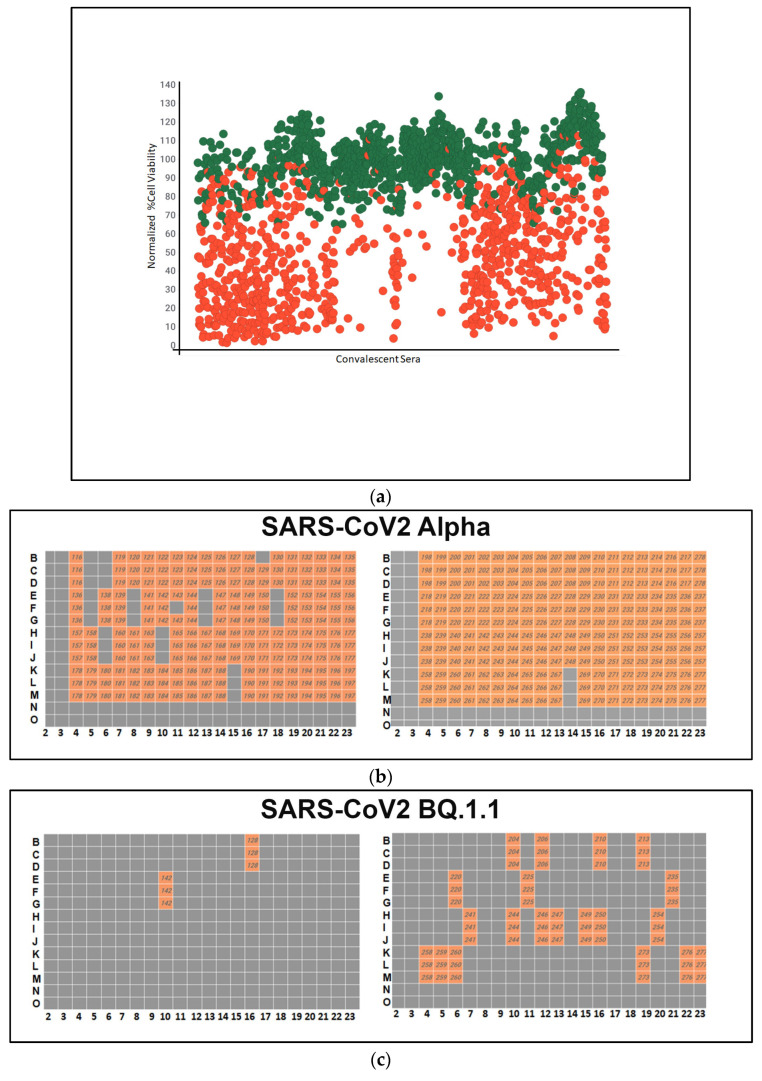

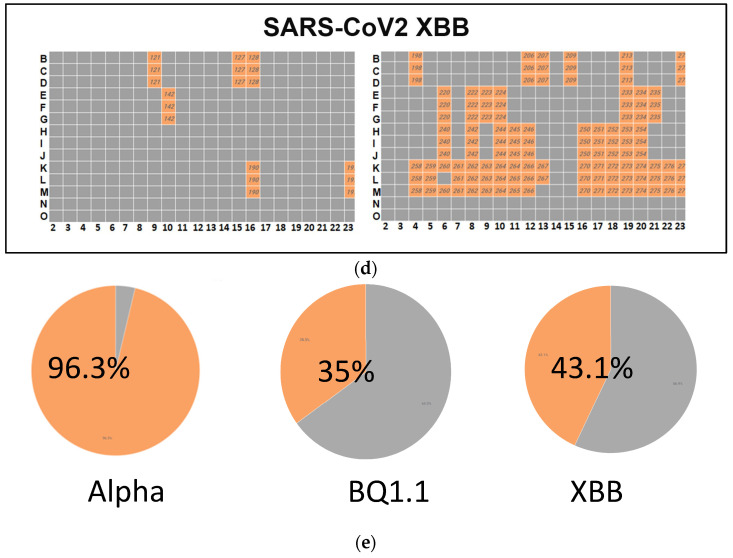
(**a**) A high-throughput primary screening assay to identify potential donors of COVID-19 convalescent serum. Using Vero-TMPRSS2 cells in 384-well imaging plates, 162 serum samples were assessed. Each sample, along with NHC, underwent triplicate screening combined with SARS-CoV-2 variants. Following a 48 h incubation, high-content imaging was used to evaluate cell viability. The Y-axis represents cell viability percentage normalized to infected control wells, while the X-axis displays convalescent serum samples. Green dots denote sera increasing cell viability by over 50%. The red dots indicate cell viability is less than 50%. (**b**) Heatmap of neutralizing Ab activity of convalescent serum samples infected with SARS-CoV-2 Alpha. Serum samples in orange-colored wells have effectively neutralized the virus, whereas those in gray-colored wells have not. (**c**) A high-throughput screening assay evaluated 162 convalescent serum samples from Columbia University Medical Center using Vero-TMPRSS2 cells in 384-well imaging plates. Each sample, alongside NHC, underwent triplicate screening against SARS-CoV-2 variants. Following a 48 h incubation, high-content imaging assessed the impact on cell viability to identify effective antibody donors. A heatmap illustrated neutralizing activity against SARS-CoV-2 BQ.1.1., highlighting successful virus neutralization in orange wells. (**d**) High-throughput screening assessed 162 convalescent serum samples from Columbia University Medical Center using Vero-TMPRSS2 cells in 384-well imaging plates. Each sample, with NHC, underwent triplicate screening against SARS-CoV-2 variants. After 48 h, high-content imaging determined cell viability. The goal was to identify sera neutralizing infections across SARS-CoV-2 variants, crucial for selecting effective antibody donors. A heatmap illustrated neutralizing activity against the SARS-CoV-2 variant XBB, highlighting successful virus neutralization in orange-colored wells. (**e**) Pie charts illustrating the effectiveness of sera in neutralizing the infectivity of samples infected with SARS-CoV-2 variants Alpha, BQ.1.1, and XBB. The percentages indicate the hit rates for each variant (orange color).

Following fixation and staining, high-content imaging was conducted to assess the impact of antiviral therapeutics on cell viability. The primary objective was to identify convalescent sera capable of inhibiting viral infection across all three SARS-CoV-2 variants. This screening process facilitates the identification of donors with the highest neutralizing antibody. The volunteers with the highest neutralizing titers may be the best candidates for the production of human monoclonal antibodies against COVID-19. The results of the screening assay showed that a large number of convalescent sera samples had high neutralizing antibody activity against the different SARS-CoV-2 variants, as shown in green in Figure 3a. The green dots on the graph represent convalescent serum samples that exhibited a more than 50% increase in cell viability compared to cells infected with the virus alone, after normalization. Each serum sample underwent triplicate testing.

A high-throughput primary screening assay to identify potential donors of COVID-19 convalescent sera was performed. A total of 162 serum samples from Columbia University Medical Center were assessed using Vero-TMPRSS2 cells in 384-well imaging plates. Each sample and NHC were screened in triplicate and combined with SARS-CoV-2 variants. Following a 48 h incubation period, high-content imaging was used to assess the impact on cell viability. The primary objective was to identify sera capable of inhibiting viral infection across all three SARS-CoV-2 variants, which is essential for identifying potential donors with effective antibodies against COVID-19. The heatmap depicts the neutralizing antibody activity of serum samples infected with SARS-CoV-2 Alpha, with orange wells indicating successful virus neutralization.

A high-throughput screening assay was used to assess 162 convalescent serum samples from Columbia University Medical Center using Vero-TMPRSS2 cells in 384-well imaging plates. Each sample, with NHC, underwent triplicate screening against SARS-CoV-2 variants. After 48 h, high-content imaging determined cell viability. The goal was to identify sera neutralizing infection across SARS-CoV-2 variants, crucial for selecting effective antibody donors. A heatmap illustrated neutralizing activity against the SARS-CoV-2 variant XBB, highlighting successful virus neutralization in orange-colored wells.

### 3.4. Dose–Response Curve and Efficacy Assessment of Individual Convalescent Serum Samples and Pooled Convalescent Sera

**Figure 4:**
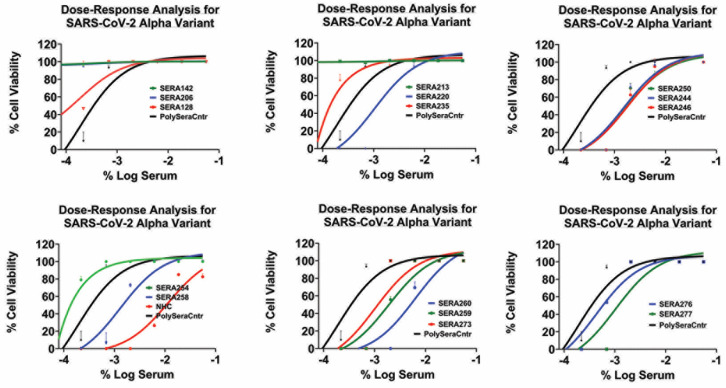
Inhibition of SARS-CoV-2 alpha infection with various convalescent sera in a dose-dependent manner. The data points represent mean inhibition percentages, with error bars indicating standard deviations. Dots above the line denote data points exceeding one standard deviation from the mean.

The dose–response graphs (Figure 4 and Supplementary Table S1) reveal the IC50 values of the convalescent sera against the SARS-CoV-2 alpha variant, ranging from 1:60 to 1:540. In comparison, the reference compound NHC exhibited an IC50 value of 0.012 μM, which is consistent with previously published data.

Importantly, none of the convalescent sera exhibited a dose-dependent response against the BQ1.1 and XBB variants, indicating their limited efficacy in neutralizing these variants. However, it is worth noting that the top two concentrations of the pooled convalescent sera (pooled sera) exhibited effectiveness against all variants of SARS-CoV-2, suggesting broader neutralization capability (Supplementary Figure S2 (HCI assay) and Figure 3 (PRNT assay)).

### 3.5. Synergy Assay

**Figure 5:**
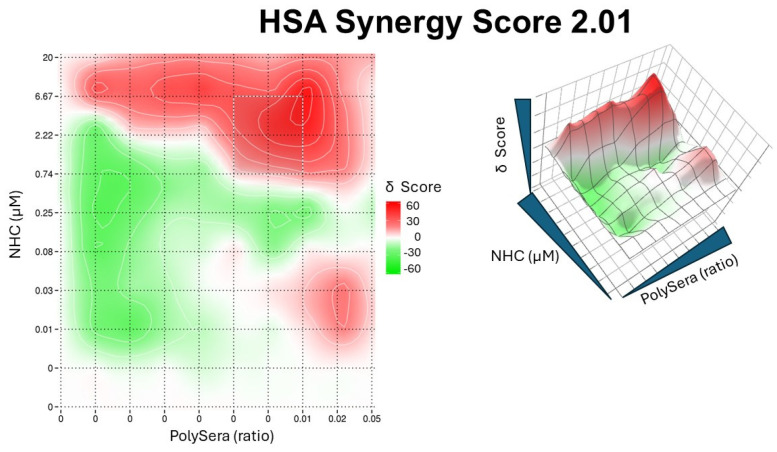
Synergy plot of pooled convalescent sera and NHC. In the left panel, the 2D topography illustrates areas of synergy. The X-axis represents the concentrations of pooled sera, while the Y-axis represents the NHC drug. In the right panel, the dose–response surface interaction combinations for pooled sera and NHC are displayed.

The results showed an absence of synergy in the studied variants, but an additive effect was observed for the Alpha variant of SARS-CoV-2, with a High Single Agent (HSA) synergy score of 2.01 (Figure 5).

## 4. Discussion

This report describes the development and optimization of *in vitro* cell-based high-throughput imaging assays for screening convalescent sera to identify suitable serum donors for use in serum therapy for SARS-CoV-2 infection in humans. In this study, we employed various cell lines, including Vero E6 cells expressing TMPRSS2, Vero-TMPRSS2, Hela-Ace2, and A542-Ace2, to identify the most suitable cell line for the initial screening assay.

Our data show that Vero-TMPRSS2 cells were highly permissive to SARS-CoV-2 infection and exhibited ease of culture, making them ideal for subsequent experiments.

Throughout the assay development stage, we meticulously evaluated multiple seeding densities of Vero cells to determine the optimal cell density that would ensure high cell viability and spatial distribution for accurate image analysis. Importantly, we observed minimal variations among replicates while maintaining precise cell segmentation. This consistency is vital for obtaining reliable and reproducible results in subsequent analyses.

We utilized an immunofluorescence assay (IFA) detection method to visualize cells infected with SARS-CoV-2. This method involved the use of a nucleocapsid-specific antibody that is conserved across different SARS-CoV-2 variants, making it well-suited for immunofluorescence assays. Our high-content imager allowed us to capture images of the infected cells, and we established specific seeding density and multiplicity of infection (MOI) values to achieve optimal infection rates. The high infection rates and robust virus replication within the cells provided an excellent platform for evaluating the efficacy of potential therapeutic antibodies that target the early stages of the virus’s life cycle.

While we extracted multiple parameters from the images to classify infected cells, our specific screening purposes in this study primarily focused on the total and infected cell counts to calculate the percentage of infected cells. This streamlined approach enabled efficient analysis and assessment of potential hits.

The robustness of our assay in the 384-well plate format was evaluated using the Z’-factor, a widely accepted measure of the separation between positive and negative controls. The calculated Z’-factor of 0.9 demonstrated statistically reliable separation, confirming the suitability of our assay for further screening purposes.

In addition to the high-content image-based assay, we developed a cell viability assay to assess the efficacy of neutralizing antibodies in preventing viral infection and preserving cell viability. By normalizing the percentage of cell viability, we established a quantitative measure to evaluate the potency of tested antibodies in neutralizing different SARS-CoV-2 variants. To ensure an accurate evaluation, we selected a specific time point of 72 h and MOIs 0.25 to 0.5 for the primary screening based on the complete loss of cells, serving as a robust reference for assessing the efficacy of neutralizing antibodies.

Our study also included a screening of a therapeutic serum repertoire against SARS-CoV-2 infections. The primary screening yielded significant hits against various SARS-CoV-2 variants, indicating the potential efficacy of the therapeutic antibody repertoire.

We compared the high-content imaging (HCI) assay alongside a traditional plaque assay (Supplementary Figure S3). This HCI assay not only demonstrated robustness but also proved invaluable because the assay uses nanoliters of samples. Hence, the HCI performed in 384-well plates spares precious samples compared to other conventional methodologies. Using HCI allowed us to perform synergy studies, which are very sample-intensive, and performing a full dose–response may not have been possible using other virological methods. Consistent with HCI assay results, the 1:40 dilution of polysera completely inhibited plaque formation by all three variants of SARS-CoV-2: Alpha, BQ 1.1, and XBB (Supplementary Figure S3). Further testing was conducted on convalescent sera at lower concentrations to assess their effectiveness before initiating dose–response studies. During the dose–response curve analysis of individual convalescent sera against SARS-CoV-2 variants, we observed varying IC50 values, indicating the neutralizing activity of convalescent sera against the SARS-CoV-2 Alpha variant. However, limited efficacy was observed against the BQ and XBB variants. Interestingly, the pooled sera exhibited effectiveness against all variants, including the recent SARS-CoV-2 variant EG.5.1 (Supplementary Figure S4), suggesting broader neutralization capability. Thus, our method may be utilized for optimizing convalescent sera cocktails for therapeutic purposes.

Our exploration of therapeutic synergy involved investigating the combined effects of multiple drugs. We found that combining polyclonal antibodies with NHC led to improved neutralization compared to individual components, highlighting the need for further investigation in this area. Cells treated with NHC alone restored cell viability to 100%, decreasing to less than 10% at the lowest concentration tested (normalized to viable Vero cells in infected wells). Serum at a 1:20 dilution alone protected cells to 100%, with efficacy lost after a 1:60 dilution, resulting in viable cells of less than 10% at the lowest dilution tested. The combination of 0.3 uM NHC and 1:60 dilution of serum increased cell viability to 47%, whereas cell viability was 23% and 22% with NHC alone or serum alone, respectively. These observations demonstrated an additive effect. Notably, only one concentration of serum at 1:60 and an NHC concentration of 1 uM showed a synergistic effect, increasing from 23% and 32%, respectively, to 75% when the drugs were used in combination. Further research is needed to explore these findings in greater detail. Similar technology can be used to accelerate the identification of the most efficacious monoclonal antibodies from B cells obtained from the serum donors. These types of methodologies should accelerate the discovery of highly neutralizing monoclonal antibodies from convalescent volunteers.

Finally our exploration of therapeutic synergy involved investigating the combined effects of multiple drugs. We found that combining neutralizing sera with NHC led to improved neutralization compared to individual components, highlighting the need for further investigation in this area.

Our research encompassed high-content image-based assays, cell viability studies, screening of therapeutic antibody libraries, dose–response curve analysis, and assessment of neutralization activity. These comprehensive findings provide valuable insights into developing effective diagnostic and therapeutic strategies against SARS-CoV-2 infections. Our work contributes to ongoing efforts to combat this global health challenge and may inform future research and interventions in the fight against emerging viral threats.

Further studies are underway to isolate B cells from serum donors whose sera had the highest neutralizing activity against SARS-CoV-2 variants. Isolating B cell clones to produce monoclonal antibodies represents an efficient approach for treating COVID-19 and other infectious diseases. This method aims to harness specific antibodies capable of targeting and neutralizing the virus, potentially offering effective therapeutic options against the disease.

## Figures and Tables

**Table 1:** Comparison of SARS-CoV-2 Variant Infection Efficiency Across Different Cell Lines.

Cell Line	Variant	Cell Number	MOI	Duration of Infection (h)	Z’-Factor
Vero-TMPRSS2	Alpha	3000	0.334	48	0.65
Vero-TMPRSS2	BQ1.1	3000	0.334	48	0.75
Vero-TMPRSS2	XBB	3000	0.334	48	0.8
HeLa-Ace2	Alpha	3000	0.5	48	0.7
HeLa-Ace2	BQ1.1	3000	0.5	48	0.88
HeLa-Ace2	XBB	3000	0.5	48	0.85
A549-Ace2	Alpha	3000	0.5	48	0.79
A549-Ace2	BQ1.1	3000	0.5	48	0.82
A549-Ace2	XBB	3000	1	48	0.82
Vero E6	Alpha	3000	0.5	48	0.9
Vero E6	BQ1.1	3000	0.5	48	0.85
Vero E6	XBB	3000	0.5	48	0.84

## Data Availability

The raw data supporting the conclusions of this article will be made available by the authors on request.

## Supplementary Materials

The following supporting information can be downloaded at: https://www.mdpi.com/article/10.3390/microorganisms12081503/s1, Figure S1: Optimizing NHC Concentration in Vero TMPRSS2 Cells for High-Content Imaging Assays for SARS-CoV-2; Figure S2: High-Content Imaging of SARS-CoV-2 Variant Infection in Vero-TMPRSS2 Cells: Assessment of Plasma Neutralization and Cellular Viability at 48 Hours; Figure S3: Micrographs depicting SARS-CoV-2 variants neutralized with Poly Antibody (pAB), variants, or untreated (mock). Figure S4: Activity of PolyAb Against SARS-CoV-2 Variant EG.5.1 in HeLa ACE2 Cells: High-Content Imaging Analysis Table S1: Table displaying IC50 values of different sera in Vero cells infected with the SARS-CoV-2 variant Alpha.

## References

[1] Cascella M., Rajnik M., Aleem A., Dulebohn S.C., Di Napoli R. (2023). Features, Evaluation, and Treatment of Coronavirus (COVID-19).

[2] Hu C.Y., Lei Y., Tang Y.W., Cui W.S., Wu P.L., Li Y.F., Zhou Y., Li X.Y., Cui H., Xiao L.S. (2023). Characteristics of patients with SARS-COV-2 PCR re-positivity after recovering from COVID-19. Epidemiol. Infect..

[3] Marcelin J.R., Pettifor A., Janes H., Brown E.R., Kublin J.G., Stephenson K.E. (2022). COVID-19 Vaccines and SARS-CoV-2 Transmission in the Era of New Variants: A Review and Perspective. Open Forum Infect. Dis..

[4] Kandula U.R., Tuji T.S., Gudeta D.B., Bulbula K.L., Mohammad A.A., Wari K.D., Abbas A. (2023). Effectiveness of COVID-19 Convalescent Plasma (CCP) During the Pandemic Era: A Literature Review. J. Blood Med..

[5] Cekmen N., Ersoy Z., Gunay Y.I., Ghavam A.A., Tufan M.Y.S., Sahin I.M. (2022). Evaluation of coronavirus diseases (COVID-19) in terms of epidemiological and clinical features, comorbidities, diagnostic methods, treatment, and mortality. J. Educ. Health Promot..

[6] Goodhue Meyer E., Simmons G., Grebe E., Gannett M., Franz S., Darst O., Di Germanio C., Stone M., Contestable P., Prichard A. (2021). Selecting COVID-19 convalescent plasma for neutralizing antibody potency using a high-capacity SARS-CoV-2 antibody assay. Transfusion.

[7] Oguntuyo K.Y., Stevens C.S., Hung C.T., Ikegame S., Acklin J.A., Kowdle S.S., Carmichael J.C., Chiu H.P., Azarm K.D., Haas G.D. (2021). Quantifying absolute neutralization titers against SARS-CoV-2 by a standardized virus neutralization assay allows for cross-cohort comparisons of COVID-19 sera. mBio.

[8] Gentleman R.C., Carey V.J., Bates D.M., Bolstad B., Dettling M., Dudoit S., Ellis B., Gautier L., Ge Y., Gentry J. (2004). Bioconductor: Open software development for computational biology and bioinformatics. Genome Biol..

[9] Aiewsakun P., Phumiphanjarphak W., Ludowyke N., Purwono P.B., Manopwisedjaroen S., Srisaowakarn C., Ekronarongchai S., Suksatu A., Yuvaniyama J., Thitithanyanont A. (2023). Systematic Exploration of SARS-CoV-2 Adaptation to Vero E6, Vero E6/TMPRSS2, and Calu-3 Cells. Genome Biol. Evol..

[10] Zhou S., Hill C.S., Sarkar S., Tse L.V., Woodburn B.M.D., Schinazi R.F., Sheahan T.P., Baric R.S., Heise M.T., Swanstrom R. (2021). beta-d-N4-hydroxycytidine Inhibits SARS-CoV-2 Through Lethal Mutagenesis But Is Also Mutagenic to Mammalian Cells. J. Infect. Dis..

[11] Kota K.P., Ziolkowska N.E., Wei J., Peng J., Ordonez D., Raney C., Prigge J., Hooper J.W., Awasthi M., Goebel S.J. (2023). Development of a rapid image-based high-content imaging screening assay to evaluate therapeutic antibodies against the monkeypox virus. Antivir. Res..

[12] Kota K.P., Eaton B., Lane D., Ulrich M., Ulrich R., Peyser B.D., Robinson C.G., Jaissle J.G., Pegoraro G., Bavari S. (2013). Integrating high-content imaging and chemical genetics to probe host cellular pathways critical for Yersinia pestis infection. PLoS ONE.

[13] Mudhasani R., Tran J.P., Retterer C., Kota K.P., Whitehouse C.A., Bavari S. (2016). Protein Kinase R Degradation Is Essential for Rift Valley Fever Virus Infection and Is Regulated by SKP1-CUL1-F-box (SCF)FBXW11-NSs E3 Ligase. PLoS Pathog..

[14] Panchal R.G., Kota K.P., Spurgers K.B., Ruthel G., Tran J.P., Boltz R.C., Bavari S. (2010). Development of high-content imaging assays for lethal viral pathogens. J. Biomol. Screen..

