# Supplementary material for: High-Throughput Screening Assay for Convalescent Sera in COVID-19: Efficacy, Donor Selection, and Variant Neutralization
Source: Microorganisms. 2024 Jul 23;12(8):1503. doi: 10.3390/microorganisms12081503 (PMC11355970; doi:10.3390/microorganisms12081503)
Supplement: Supplementary file 1 [file microorganisms-12-01503-s001.zip › microorganisms-2908797-supplementary.pdf]

Supplementary Data

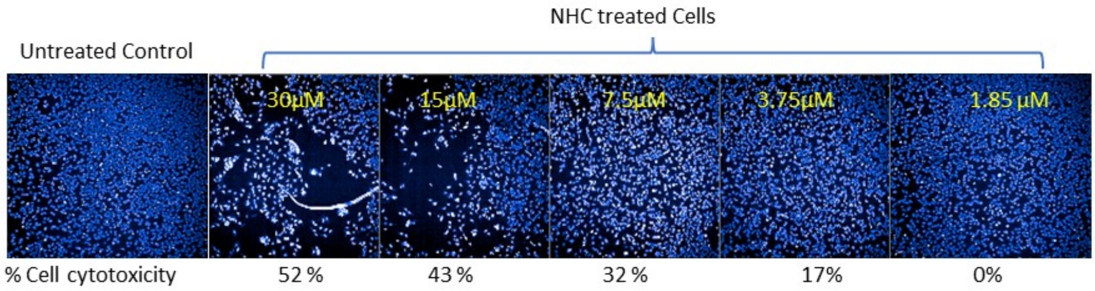

Figure S1. Optimizing NHC Concentration in Vero TMPRSS2 Cells for High-Content Imaging Assays for SARS-CoV-2: Fine-tuning the NHC concentration in Vero TMPRSS2 cells to establish the optimal level for serving as the positive control in high-content imaging assays for SARS-CoV-2. Blue represents cells treated with Hoechst, which highlights the nuclei

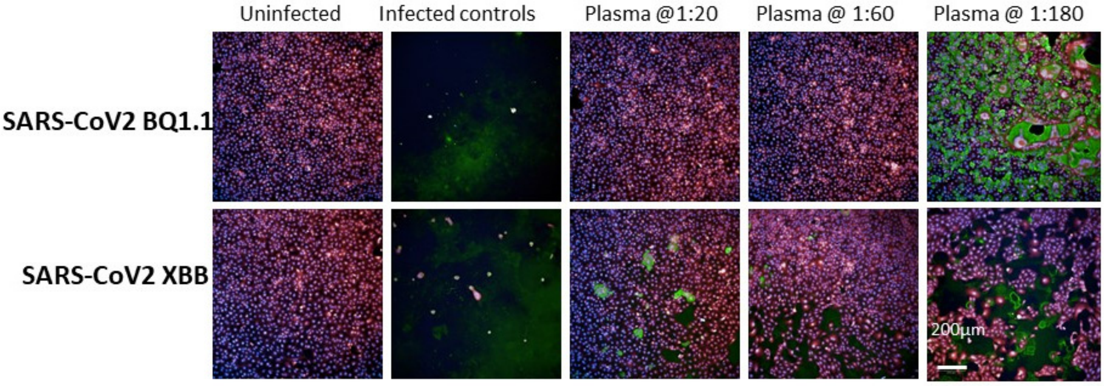

Figure S2. High-Content Imaging of SARS-CoV-2 Variant Infection in Vero-TMPRSS2 Cells: Assessment of Plasma Neutralization and Cellular Viability at 48 Hours: High Content Images display Vero-TMPRSS2 cells infected with two SARS-CoV-2 variants for 48 hours post-infection, employing a multiplicity of infection (MOI) of 0.37. Preceding infection, the virus underwent neutralization for 1 hour using plasma, with concentrations serially diluted threefold, commencing at 1:20. The viability of Vero-TMPRSS2 cells was verified to be ≥ 80% in the top two plasma dilutions compared to infected cells 48 hours post-infection. Within these high-content images, virus-infected cells are delineated in green, the entire cell population is depicted in red using Cellmask, and the nuclei are stained blue with Hoechst dye

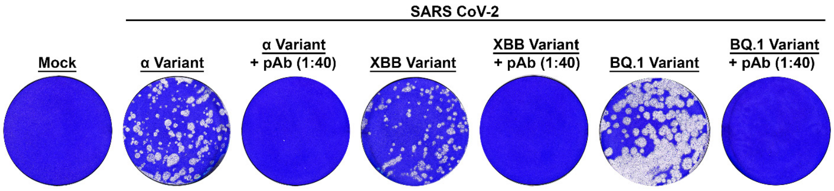

Figure S3. Micrographs depicting SARS-CoV-2 variants neutralized with Poly Antibody (pAB), variants, or untreated (mock) .

To get further confirmation on the neutralization activity of the antibodies, PRNT 50 assays were performed against three SARS-CoV-2 variants; Alpha, XBB, and BQ1. The monoclonal antibodies were pooled to generate an antibody cocktail and diluted to 1:20 and 1:40 in the PRNT 50 assay. Mock-infected and virus-infected wells were used as negative and positive controls, respectively. All virus-infected wells had virus plaques. In contrast, all wells the cocktail antibody and virus variant mixtures at 1:20 (data not shown) and 1:40 dilutions exhibited no plaques. These data demonstrate the potent neutralizing activity of the antibodies and verify the results of the screening study.

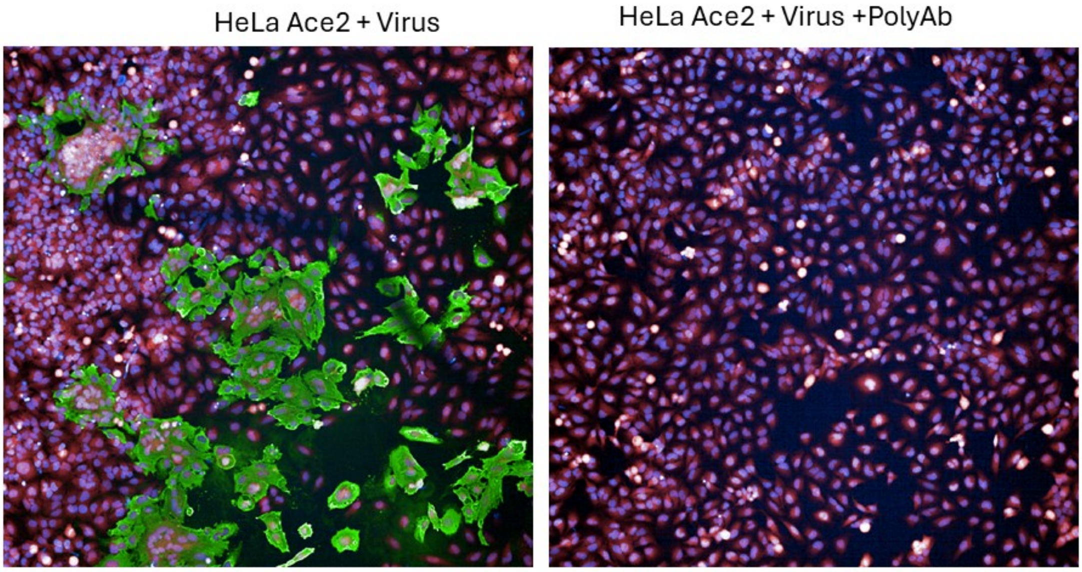

Figure S4. Activity of PolyAb Against SARS-CoV-2 Variant EG.5.1 in HeLa ACE2 Cells: High-Content Imaging Analysis PolyAb exhibits activity against the SARS-CoV-2 variant EG.5.1 in HeLa ACE2 cells. In these high-content images, virus-infected cells are highlighted in green, while whole cells are stained in red using CellMask™, and nuclei are stained in blue using Hoechst dye

Table S1. Table displaying IC50 values of different sera in Vero cells infected with the SARS-CoV-2 variant Alpha

Serum	IC50 Value
SERUM128	0.000186
SERUM142	0.000295
SERUM206	0.000372
SERUM213	0.001461
SERUM220	0.001037
SERUM235	4.63 E-09
SERUM244	0.00151
SERUM246	0.001751
SERUM250	0.001643
SERUM254	4.41-E-09
SERUM258	0.001309
PooledSeraCntl	0.000203
NHC	0.01153
SERUM259	0.0001888
SERUM260	0.006262
SERUM273	0.001024
SERUM276	0.000429
SERUM277	0.0001024
